# Factors that influence the sustainability of structured allied health journal clubs: a qualitative study

**DOI:** 10.1186/s12909-018-1436-3

**Published:** 2019-01-03

**Authors:** Rachel Wenke, Katherine O’Shea, Jo Hilder, Rae Thomas, Sharon Mickan

**Affiliations:** 1 Allied Health Clinical Governance, Education and Research, Gold Coast Health, Gold Coast University Hospital, 1 Hospital Boulevard, Southport, QLD Australia; 2 School of Allied Health Sciences, Griffith University, Gold Coast, QLD Australia; 3 Centre for Research in Evidence-Based Practice, Faculty of Health Sciences and Medicine, Bond University, Gold Coast, QLD Australia; 4 Occupational Therapy, Faculty of Health Sciences and Medicine, Bond University, Gold Coast, QLD Australia

## Abstract

**Background:**

Structured journal clubs are a widely used tool to promote evidence-based practice in health professionals, however some journal clubs (JC) are more effectively sustained than others. To date, little research has provided insights into factors which may influence sustainability of JCs within health care settings. As part of a larger randomised controlled study, this research aimed to gain understanding of clinicians’ experiences of sustaining a structured JC format (TREAT- Tailoring Research Evidence and Theory) within their clinical context. The study also aimed to identify which strategies may assist longer term sustainability and future implementation of the TREAT format.

**Methods:**

We employed a qualitative methodology, informed by behaviour change theory. Clinicians (*n* = 19) from five different JCs participated in focus groups to explore their experience in sustaining the JC format six months after the formal trial period had completed. Clinicians were asked to describe factors which they perceived helped or hindered sustaining components of the JC format within their local context. Following a descriptive summary of the data, barriers and enablers were thematically analysed according to behaviour change theory domains: capability, motivation and opportunity and further mapped to targeted implementation strategies.

**Results:**

Participants reported perceived benefits of maintaining the TREAT format and described several components that promoted its sustainability. Sustaining factors linked to individuals’ capability included building research knowledge and skills and having access to research experts. Sustaining factors that enhanced opportunities for behaviour change included management expectation to attend and a team culture which values evidence based practice, while factors found to enhance individuals’ motivation included the JC having close application to practice and clinicians sensing ownership of the JC. Several implementation strategies to enhance these factors are described including graduated support to clinicians in facilitation of JCs and greater engagement with managers.

**Conclusions:**

Long-term sustainability of a structured JC is dependent on both individual and service level factors and a balance of implementation strategies that enhance capability, opportunity and motivation. Consideration of how clinicians can be engaged to take ownership and build their own capability from the commencement of the JC is important.

**Trial registration:**

ACTRN12616000811404.

**Electronic supplementary material:**

The online version of this article (10.1186/s12909-018-1436-3) contains supplementary material, which is available to authorized users.

## Background

While evidence-based practice (EBP) has significant benefits to patient care, the majority of health professionals experience challenges implementing EBP in their everyday practice. Allied health professionals (AHPs), who are comprised of dietitians, occupational therapists, physiotherapists, pharmacists, psychologists, speech pathologists, and social workers, among others, frequently report that reduced time, skills and confidence hinder their ability to implement EBP in daily practice [1, 2]. Journal clubs (JC), where health professionals meet to appraise and discuss a journal article, are a widely used tool which may increase EBP skills and facilitate the uptake of evidence in clinical practice [3–7]. AHPs report variable experiences about the usefulness of JCs [5] and few studies have purposefully focused on allied health, with the majority of research using case-based [7–9] or uncontrolled designs [6].

Evidence from medical literature has highlighted several key components which may enhance the effectiveness of JCs in promoting EBP skills within allied health [3, 4] including using goal setting, formal facilitation, adult learning principles, support from researchers and a critical appraisal tool. These components however are often not incorporated in allied health JCs, whereby traditionally an AHP chooses a research article of personal interest and talks about it without using any specified structure [5]. The introduction of more structured JCs has recently been explored in the literature. For example, Lizarondo et al., [6] evaluated the effectiveness of a structured JC format in 93 AHPs from five professional groups (i.e., physiotherapy, speech pathology, nutrition, occupational therapy and social work) within South Australia. Clinicians were provided with pre-appraised research evidence from external academic support. Following the six month trial, Lizarondo et al., reported significant improvements in objective and self-reported measures of EBP knowledge, with some professional groups also self-reporting increased evidence uptake and improved attitudes towards EBP [6].

More recently, Wenke, Thomas, Hughes and Mickan [10] undertook the first randomised controlled trial evaluating the impact of a structured JC format Tailored according to Research Evidence and Theory (TREAT) within a large tertiary health service in Queensland, Australia. Nine JCs with 126 allied health participants were allocated using clustered randomisation to receive either the structured TREAT JC format or the standard JC format for 1 h/month for 6 months. The TREAT format incorporated eleven key components of successful JCs as evidenced in two systematic reviews, as outlined in Table 1 [3, 4]. For example, in contrast to the standard format, TREAT JCs used initial goal setting, group-based appraisal using freely available Critical Appraisal Skills Programme (CASP) tools [11] (with a tool chosen dependant on the research design of the journal article) and formal facilitation by an academic mentor. Following the six-month trial, participants receiving TREAT were significantly more satisfied compared with the standard format, however measures of skill, knowledge and attitude did not differ between groups. This may have been a result of ceiling effects and reduced sensitivity of measures, as clinicians reported qualitative changes in their confidence and skills in undertaking EBP [10].

**Table 1:** Components of TREAT Journal Club Format

Component from evidence	Consistently conducted in standard JCs?	Description of local application
1. Establish JC of similar interests ^+^	✔	- JC participants from similar clinical background or interest - Initial goal setting session to establish topics of interest to all members that will be discussed in journal club.
2. Have overarching goal and purpose ^+^	☓	As above.
3. Regular predictable attendance ^+^	✔	Journal club set at same time and location each month
4. Circulating articles for discussion ^+^	☓	Journal articles circulated prior to journal club
5. Didactic support ^+^	☓	Didactic teaching initially provided within each session on given topic by research academic and later given as handouts for reference
6. Mentoring/Support from researchers/academics ^+a^	☓	Academic facilitator available for support between sessions
7. Have a facilitator to guide discussion^+^	☓	Academic facilitator helped guide discussions during each session
8. Use of structured appraisal tools during the session ^+a^	☓	Standardised critical appraisal tool used “Critical Appraisal Skills Programme” or CASP.
9. Adhering to principles of adult learning and use multi-faceted learning strategies^a^	☓	-Group approach to critical appraisal to promote collaborative learning-Incidental teaching based on participant motivations within the session- Written based resources and access to library support to assist with searching
10. Put evidence in context of clinical practice and evaluate knowledge uptake informally or formally ^+^	☓	Time provided in session to discuss clinical implications and follow up of knowledge uptake.
11. Provide food^+^	☓	Journal club participants invited to bring food to share for session

^**+**^= key component suggested in Deenadayalan et al., 2008 ^a^ = key component suggested in Harris et al., 2011

Together these studies suggest positive benefits of using structured JCs in allied health, for both feasibility and using research to influence clinical practice. It is unclear however whether these structured JC formats can be sustained within busy clinical contexts, and what helps or hinders their sustainability. When implementing any innovation it is important that sustainability of that intervention is considered early so that the outcomes of the initiative within the local context are sustained beyond the “trial period”, however most innovations rarely consider such in their design [12]. Indeed, a recent systematic review broadly evaluating how interventions within healthcare are sustained encouraged researchers to consider sustainability when implementing new programs to help understand why and how some interventions last and others do not [13].

To date, there has been little investigation into the factors which influence sustainability of JCs within allied health [5]. Lizarondo et al. in a separate qualitative study [5] explored the views of South Australian AHPs regarding JCs in promoting EBP and evidence uptake in the workplace. Although long term sustainability was not a focus of the enquiry, participants reported that limited knowledge of statistics and a heavy clinical workload were key barriers impacting long term sustainability of JCs, while mentoring and use of professional development points as incentives to participation were enablers. Knowledge from different contexts is needed to understand how local factors can influence longer term uptake of components of JCs in different clinical settings. Behaviour change is a key construct of sustainability [14], and the use of a behavioural change theoretical framework [15] may further assist in understanding sustainability. For this study, details of the local clinical context were analysed using a behaviour change framework to guide development of future implementation strategies for JCs that may influence AHP’s subsequent use of EBP in everyday practice. Use of a behaviour change theoretical framework helps to design and select interventions that can be linked to the constructs of a targeted behaviour [15].

## Method

### Aim of study

As part of a larger randomised controlled study evaluating the effectiveness and feasibility of the TREAT JC format [10], we aimed to better understand clinicians’ experiences of sustaining the TREAT JC format at six-months post-intervention, both generally within their clinical context as well as in sustaining the different components of the TREAT format.

Specifically, we sought to answer the following research questions,

1. What were clinicians’ overall experience in sustaining the TREAT JC format within their clinical context and what helped or hindered this format’s sustainability?
2. What were clinicians’ experience in sustaining each of the eleven core components of the TREAT format and what helped or hindered the sustainability of these components?
3. What strategies and adaptations might assist with the longer-term sustainability and future implementation of the TREAT format within an allied health workforce?

### Study design and setting

This study employed a qualitative methodology, with analysis and interpretation informed by behaviour change theory. The research was conducted within hospitals and community centres of a large non-metropolitan governmental health organisation. Ethical approval for the study was provided (HREC/15/QGC/310) prior to commencement.

### Study participants

A total of 61 participants from five different existing JCs participated in the TREAT JC format described in Wenke, Thomas, Hughes and Mickan [10]. This included 52 AHPs and nine nurses. Using purposive sampling to facilitate even representation across allied health professions and clinical experience, 28 AHPs from the TREAT JC intervention group were invited via email to participate in focus groups six months after completing the intervention trial.

### Intervention

TREAT (Tailoring Research Evidence and Theory) JCs comprise 11 key components evidenced in the literature to be active ingredients for effective JCs (see Table 1) [3, 4]. These components were tailored to meet the needs of the local health care context. All JCs met for one hour each month for six months. The TREAT JCs were facilitated by an academic who was experienced in teaching EBP. For the final session, a volunteer clinician from the JC co-facilitated the session with the academic facilitator’s guidance. For further details and resources regarding running of this JC please refer to our previously published paper [10]. Following the six-month intervention period, participants in the TREAT JC were provided with resources to continue the TREAT format including session format guides, presenter and facilitator guides, and minute and critical appraisal templates. No other formal facilitation from the academic mentor was provided during the follow up period however clinicians were free to connect with other researchers and/or academics within their existing networks to assist with the JC, particularly critical appraisal of the journal article if required.

### Data collection

Six months after the intervention, a purposive sample of participants from each TREAT JC were invited to participate in one of six follow up focus groups with other members of their own JC. Interview questions were designed to explore participants’ experiences in maintaining the TREAT JC format by identifying factors which helped or hindered sustainability. Questions also specifically addressed all 11 key components [3, 4]. Probing questions about barriers and enablers relating to participants’ capability, opportunity or motivation to sustain the intervention were included in relation to the COM-B model of behaviour change theory [15]. The COM-B model was developed from a synthesis of many theories of behaviour change [15], and proposes that people need capability, opportunity and motivation to perform behaviour. For example, for someone to engage in a specific behaviour, they must have the physical and psychological capability, within appropriate social and psychological opportunities and be able to want to, or need to do the behaviour more than any other competing behaviours at the time. Participants were sent a copy of the interview questions via email one week prior to the interviews (Additional file 1). Two facilitators (KO and JH) conducted the interviews using the same semi-structured interview guide. Both facilitators were health professionals with a research and/or education background, who were not involved in providing the TREAT JC format, nor supervising any of the participants. The focus groups were conducted at a mutually convenient location within the clinician’s workplace and lasted for approximately 45- 60 min. Each interview was audio recorded and the facilitator took field notes during the interviews.

### Data analysis

A professional transcription service transcribed all audio interview recordings. Two researchers, RW and KO, used NVivo 10 [16] to independently code transcripts into categories and sub-categories to provide a descriptive summary of the data. To answer the research questions, categories and sub-categories were coded using an initial agreed coding framework which included: general experience, contextual enablers and barriers, recommendations and specific barriers, enablers, barriers and adaptations for each core component of the TREAT format. Discrepancies between the researchers were discussed until a consensus was reached.

Following the descriptive analyses, KO and RW analysed the identified barriers and enablers with reference to the COM-B model. For this research project, the COM-B model was used to analyse the qualitative data to better understand and explain barriers and enablers, and to answer our third research question in identifying future strategies for implementation. This level of analyses included mapping each barrier and enabler to one or more of the three constructs of the COM-B model (1) capability (2) opportunity or (3) motivation. An additional level of synthesis occurred within each construct, to identify key themes. Where enablers or barriers were mapped to more than one of the COM-B constructs, the most salient construct for each item was identified to reduce redundancy. To identify relevant implementation strategies which address the barriers and enablers within motivation, capability and opportunity, the Miche et al., [14] methodology was followed. These barriers and enablers were linked to one of eight behaviour change intervention functions that are likely to be effective in bringing about desired change in the target behaviour.

## Results

### Participants

Thirty JC members were invited to attend a focus group and ten declined because they were not available at the allocated times or did not respond (*n* = 1). Nineteen health professionals consented to participate in the focus groups. Participants represented five different TREAT JCs as shown in Table 2 and included predominately females (*n* = 16). Due to the JC 2 being across two sites, two focus groups were undertaken for this JC. Only 1 participant attended for JC 4 because of staff rotations at the end of the intervention period. Participant demographics and professional groups are reported in Table 2. Six-months after the intervention had completed, two of the five JCs, reported to have sustained the majority of the components of the TREAT format for the entire period, with plans to continue the format indefinitely. Of the remaining JCs, two had between two and three JC meetings since the intervention and one JC did not meet at all. All participants however reported intentions to either continue with, or to re-instate JCs within their current practice setting using elements of the TREAT format. The clinicians’ experiences in sustaining the TREAT format were summarised in relation to four categories as shown in Table 3.

**Table 2:** Participant characteristics

Participant details (*n* = 19)	Participants in original JC (*n*=)	Participants invited to focus group	% of original JC participants in focus group (*n*=)
Focus group participants			
Journal club 1 (Community, MDT)	15	4	15.8 (3)
Journal club 2 (single profession, inpatient)	21	12	42.1 (8)
Journal club 3 (single profession, inpatient)	9	3	15.8 (3)
Journal club 4 (single profession, inpatient)	7	3	5.2 (1)
Journal club 5 (Community, MDT)	9	7	21 (4)
Profession			
Psychology			10.5 (2)
Occupational Therapy			21 (4)
Dietetics			52.6 (10)
Physiotherapy			10.5 (2)
Podiatrist			5.2 (1)
Clinical Experience			
Base grade clinicians (entry level)			31.5 (6)
Senior health clinicians			68.5 (13)

MDT = Multidisciplinary team

**Table 3:** Summary of descriptive analyses & frequency of mention

Theme	Subtheme	Freq. Mention^a^
Perceived benefits of TREAT	Perceived positive value or improved/easier	21
	Increased knowledge and skills	19
	Improved structure and organisation	12
	Increased interaction	9
Contextual Enablers	TREAT and EBP experience within own team	9
	Work unit/leadership culture values EBP	7
Contextual Barriers	Competing demands deprioritise JC	21
	Planned and emergent staffing changes	11
	Perceived lack of confidence and capability	10
	Video conference engagement	4
	Reduced external accountability	4
Clinician Recommendations & Future Plans	Build internal capacity and ownership	9
	Ongoing involvement of academic mentor	6
	Further EBP and stats training	4
	Integration of EBP in everyday practice	4
	Changes to TREAT format	2

^a^This refers to the number of different times this category was mentioned within the focus groups

### Perceived benefits of the TREAT JC format

Across all focus groups, clinicians described benefits of the TREAT format compared to their previous JC experiences. For example, participants reported overall greater value in the TREAT format compared to previous JCs, *“it’s been a positive change and most people are getting more out of the journal clubs”* [F2]. Clinicians also reported perceived improvements in their knowledge and skills, “*there’s personal learnings that I’ve taken about the way of approaching that evidence-based practice aspect of my work*” [F6]. Clinicians perceived the structure and organisation of the TREAT format to be beneficial compared to previous JC formats, as one clinician described *“prior to that we would have just been randomly picking an article and doing whatever we did with it”* [F2]. The increased interaction introduced by the TREAT format was also described favourably, *“I think it’s a lot better than … it was 12 months ago when you were just sitting there listening to a PowerPoint presentation and there was really no interaction at all”* [F2].

### Contextual enablers

Participants identified two key enablers to sustaining the TREAT format within their local contexts. One enabler was clinicians having research and EBP experience within the team to support the JC *“we’ve always had a mentor, we’ve always had somebody stronger in the research practice”* [F1]. Clinicians with previous exposure to the TREAT format were able to lead the continuation of the structured format, as one clinician described, “*There’s probably a lot of long standing staff members here that will still drive that [TREAT format]”* [F2] Moreover, when clinicians had previous experiences in applying EBP skills such as critical appraisal, this was also seen to positively influence continuing the format, *“I don’t think anyone is coming in without any skills in the area because we critically appraise during university, it’s a big component”* [F2].

Clinicians described when their leaders reinforced the value of using research evidence to inform practice, the continuation of the JCs was easier, *“So our team leader is very pro saying, this [JC] is... important, so this is part of your professional development. So it’s like, why aren’t you turning up....rather than, can you please turn up”* [F1].

### Contextual barriers

Several contextual barriers were identified as influencing the JC’s sustainability. Clinicians reported that competing demands in their caseloads led to them de-prioritising JC attendance, *“I think it’s just the inevitable conflicting time issues when you’ve got a clinical workload and that’s why sometimes in our department we don’t get full attendance but that’s not going to change” [F2].* Staffing changes also negatively affected continuation of the JCs *“For six months post [intervention] we lost a lot of staff and so the skills weren’t translated”* [F4].

Participants’ perceived lack of confidence and capability in their own EBP skills also had a negative impact on continuing the format, “*It’s really difficult to critically appraise the statistical analyses component because we didn’t have that subject matter expertise*” [F6]. The difficulty of staff from different geographical work units meeting together and the technical challenges of using video conference facilities was also perceived as a barrier, *“I think the engagement .. is an issue for lots of the times, especially if it’s via [video conference] So people sometimes get confused to what other people are talking about. I think this is a facilitated learning process, if you don’t get that face-to-face interaction, it loses its power a little bit”* [F3].

Impressions of a lack of external accountability for continuing their attendance also affected sustainability, for example clinicians felt that while management endorsed JC practice, it was not considered part of their core business, *“management endorses … JC and they say yeah, it’s a good thing. Go do it. Just make sure you do everything else as well and our patients are our number one priority, that’s our core business”* [F6]. Another clinician described a lack of accountability for attending JCs, “*It’s left to [my] …*. *own devices, basically.*” [F6]. Consequently, it was difficult to align competing priorities for all staff and when there was limited accountability in departments for JCs, at times JC were cancelled or rescheduled, “*if we did have things organised they often got cancelled because there was no other sort of external accountability”* [F4].

### Sustainability of individual components of TREAT format

The participants described how the individual components of the TREAT format were sustained, as well as barriers and enablers to their sustainability. The COM-B [15] was utilised to better understand how barriers and enablers influenced participants’ perceptions of their capabilities, opportunities and motivations.

### Components most readily sustained

Table 4 outlines the seven individual components of the TREAT format that were described by clinicians as most readily sustained. Having a process already established prior to the TREAT format of circulating articles prior to the session was seen as an enabler to sustaining this component, as one clinician indicated *“The articles were always circulated before (TREAT)”* [F2]. The regular use of structured critical appraisal frameworks (e.g., CASP tools [11]) were also seen as helpful, *“the structured tools …*. I *definitely find that they were helpful and that’s something that is definitely continued on within the JCs”* [F4], however knowing which tool to use was considered a challenge for some clinicians*.* Having consistency in the time and place of the JC meetings was a component of the TREAT format that was important, *“the club at the same time and place because it was during the meeting times. So that has always been sanctioned”* [F4]. The participants spoke frequently of the ongoing benefit of discussing the application of the evidence within the JC, including, *“I think at the end of our journal [clubs] we will still discuss whether it would impact on work here and whether we make any changes...”* [F2]*.*

**Table 4:** Individual components of TREAT format most readily sustained and barriers and enablers to sustaining mapped to COM-B model [10]

Component	Barriers to sustaining	Enablers to sustaining
Articles circulated prior	• Competing demands, often sent late (O)	• Articles previously circulated before TREAT format introduced (O) • Sense of responsibility to send early (M)
CASP tools	• Knowledge which specific study design CASP tool to use (C)	• Having confident clinician help choose tool (C) • Perception that tool makes appraisal more accessible & guides session (C) • CASP tool structure helpful (C)
Consistent time and place	• Less regular scheduling/cancellations/emergent leave (O) • Unprepared presenters (O) • Difficulty finding time suits everyone(O) • Less accountable without academic (O)	• Set time allocated (O) • Clinician encouraging attendance just prior to meeting (M) • Manager expectation (M) • Having JC after another set meeting (O)
Discussing applying evidence	• Difficulty applying to practice for multi-disciplinary team (C) • Can’t always change practice from article (O) • Lack of confidence in applying evidence (C)	• Greater understanding of other multidisciplinary team roles with JC (M) • Members all contribute to discussion (M) • Senior staff present to assist with application to practice (C)
Group appraisal	• Reduced quality of appraisal without academic (C)	• Less intimidating when appraisal done as a group (M) • More participation in group discussions than previous format (M) • Split into smaller groups (O)
Librarian support^	• Lack of awareness or how to access (C)	• Quick turnaround to receive articles (O) • Perception library can help find higher quality article (M) • Helpfulness of librarians (M)
Food at meetings	• Perception that motivation should be intrinsic (M) • Cost of food (O)	• People bring own food/chocolate (O) • Motivates people to attend (M)

Note: (*M*) Motivation component of COM-B, (*O*) Opportunity component of COM-B, (*C*) Capability component of COM-B. *CASP* Critical Appraisal Skills Programme

The participants also described the group appraisal component as being a factor they were continuing to use, “*… we’re still doing great discussions and I guess maybe sectioning the questions to different group [members] and then coming back as a group to give the answers”* [F2]. A barrier to sustaining this group-based appraisal was the lack of a research mentor present, *“without having the facilitator there - I think the biggest things that will be affected are that group discussion and a really thorough discussion of how evidence can be applied in the clinical setting”* [F4]. Librarian support was able to be sustained because of the helpful nature of library staff, “*They’re just incredibly helpful. They will just help you to understand what it is you actually want, and do it for you, essentially”* [F5].

### Components most difficult to sustain

Table 4 provides a summary of the five individual components of the JC format reported as most difficult to sustain, along with barriers and enablers, and adaptations that were used to sustain or support the component. Several barriers were reported to sustaining an academic mentor’s presence as part of the JC format includingreduced confidence in their absence, *“coordinating the flow of looking through an article, and actually feeling confident with their interpretation from the article had been quite hard, since [academic facilitators] stopped coming*” [F5]. As an adaptation to this component, clinicians identified colleagues with increased EBP experience and suggested they could actively support their team, *“Then I would be able to go to [clinician name] and say, show me...how to do this and then I’ll be able to do it”* [F1]. The role of a formal facilitator in the JC was also adapted so that the presenting clinician assumed this role, *“well, usually the person that’s presenting the article (facilitates) and you (clinician) support.”* [F1]. Similarly, the topic selection (i.e., goal setting) component was adapted so that the presenter set the topic for each JC rather than the topics being chosen collaboratively by the group in advance.

Neither educational handouts nor minutes were sustained with one clinician commenting, *“I’m wondering how much time would be involved to prepare those PowerPoints and the handouts … that’s additional work which may present as a barrier”* [F6]. As an adaptation to taking minutes, clinicians from one of the JCs reported that the presenting clinician was assumed to follow up actions, *“the people who have actually selected that article they will generally follow that up*” [F2], however this did not include a written record.

### Clinician recommendations and future plans

Participants across all JCs generated some recommendations and reported some plans for future JCs. Building internal capacity and ownership was recommended consistently across a number of JCs, for example *“maybe embed like an apprenticeship model where...a couple of people who self-identify as being really keen to extend their skills in this area - do some extra training”* [F4].

Ongoing access to an academic mentor or expert in JCs was recommended, for example, “*… having one of the facilitators come every few months maybe just to reinforce that we’re on the right track or to come back and explain some of the statistics and things that we’re not as confident with”* [F2]. Clinicians recommended further training, especially “*…*. *in relation to research, like, how to read stats, how to understand stats, what are we looking for, what’s important in this”* [F3]. Clinicians also recommended further integration of research evidence into their everyday practice, for example “*… It’s got to appeal to what they need to learn and what they’re doing in their day-to-day work”* [F6]. One example was to incorporate the use of JC to “*help … with different quality improvement activities”* [F5].

Lastly, participants made recommendations relating to the structure of the TREAT format, including extending the length of time, *“I just don’t think that the six months was long enough considering it’s really only six sessions and with the amount of staff rotations it’s hard to embed within the service”* [F4] and having greater time to discuss the application of evidence into practice in the sessions was also suggested, “*I think there’s a really important component and that is the kind of knowledge translation into practice and I’m not sure if we’re actually getting that component of it happening.”* [F6].

#### Analysis of sustainable behaviour change and strategies

**Fig. 1:**
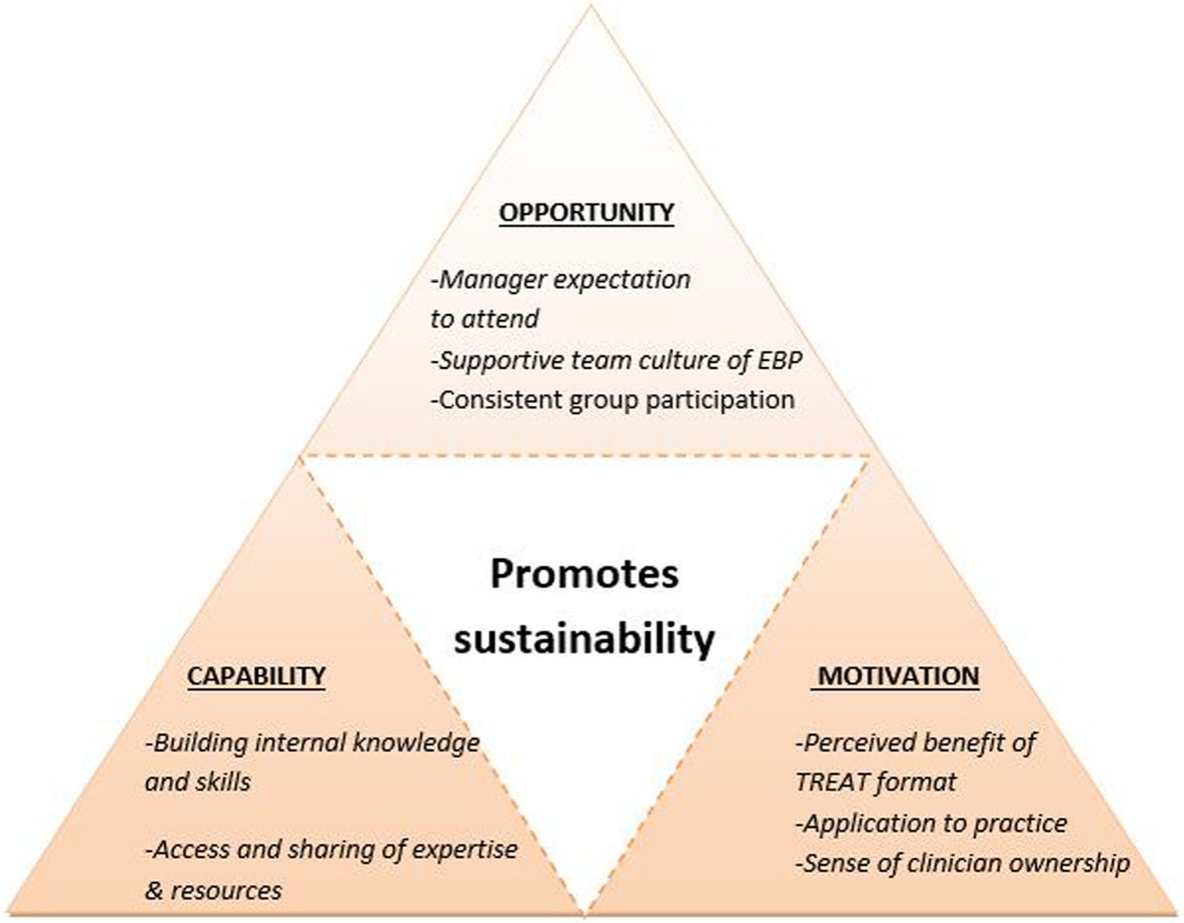
Schematic diagram of factors which influence sustainability

The secondary analyses synthesised the barriers and enablers described in Tables 3-5 into the three constructs of the COM-B model. Subthemes under each of these three constructs are presented in Table 6, which were used to inform implementation strategies to target the identified barriers or to enhance the enablers. Enablers and barriers were mapped across all three constructs of the COM-B, however the most prevalent domain was related to ‘opportunity’. The majority of implementation strategies involved intervention functions of enablement, modelling or education and persuasion that according to Michie et al., (2014) were most likely to bring about (or sustain) behaviour change. For example, a barrier identified to influence motivation to sustain the JC was related to clinician’s perception of the benefit of the TREAT format. An implementation strategy to address this barrier which uses persuasion and incentivisation to enhance motivation could be for clinicians familiar with the TREAT format to share their positive experiences with other members. The subthemes of Table 6 were further synthesised in Fig. 1 which reveals key factors which promote sustainability.

**Table 5:** Individual components of TREAT format most difficult to sustain and adaptation strategies

Component	Barriers to sustaining	Enablers to sustaining	Adaptations
Academic mentor present	• Reduced awareness of how to access academic (C) • Expense having academic every session (O) • Finding researcher with appropriate expertise (O) • Academic’s knowledge intimidating (M) • Less confident without an academic (C)	• Having another researcher in department to help (O) • Increased accountability to attend (M)	• Clinicians supporting EBP skills
Facilitator guiding discussion	• Need expertise in statistics (C) • Videoconference impacts discussion (O) • Facilitation more difficult in absence of academic (C)	• Having clinician guiding discussion (O)	• Presenter does facilitation rather than separate role
Goal setting	• Topics less relevant after 6 months (M) • Difficulty coming up with relevant or important topics, particularly new grads (C) • Resistant to change and lack of ownership (M) • Not having academic to assist (C)	• Choosing current topics rather than six months in advance (M) • Using TREAT step by step resource (O) • Support from line manager (M) • Choose topics relevant to practice (M)	• Clinicians just choosing topics individually that are interesting to them • Talk to clinicians in team with more confidence in JC for guidance
Educational handouts provided	• Time burden (O)		
Minutes & formal follow up	• Perceived lack of benefit (M)	• Keep people accountable to following up items (M) • Perception of useful record of discussion (M) • Technology supports to do minutes online (O)	• Give brief updates on application of previous JCs at next JC • Person presenting follows up action regardless of minutes

Note: (*M*) Motivation component of COM-B, (*O*) Opportunity component of COM-B, (*C*) Capability component of COM-B

**Table 6:** Factors which influence and enhance behaviour change to implement JCs

COM-B Domain and associated factor	Behaviour Change Wheel Intervention Functions	Implementation strategies
Motivation		
Perceived relevance of topics linking to clinical practice	Enablement Environmental restructure	• Ensure group engagement during prioritisation of topics (consider topics that relate to current clinical service priorities or Quality Improvement projects) • Longer time dedicated to discussion of application of evidence in each session. • Skilled JC member facilitates discussion
Clinician ownership, sense of responsibility & accountability	Modelling	• Identify 2–3 clinicians to co-facilitate JC and holder of “JC portfolio” • Presenting clinicians to follow up the action items & feedback at future JC sessions
Perceived benefit of format	Persuasion Incentivisation	• JC members familiar with TREAT to share positive experiences with others
Belief that participation in JC improves knowledge and skills in EBP	Persuasion Education	• Use of positive experience stories to encourage belief that capability increases with ongoing JC attendance and engagement
Other clinicians encourage attendance prior to meeting	Persuasion	• Use of email reminders prior and electronic reminders in electronic calendars to prompt attendance and reduce double-bookings
Opportunity		
Staff changes including planned changes (eg rotations, planned leave & staffing availability) & emergent leave (eg sick leave & workforce shortages)	Education Training	• Consider upskilling non-rotational staff to improve resilience during rotations (i.e. upskilling senior staff who do not rotate) • Increase number of staff exposed to JC, in order to saturate skills across the workforce
Competing demands leading to JC deprioritised due to clinical demands	Environmental restructuring Enablement	• Timetable of presenters with consistent time and place booked in clinicians’ calendars (updated electronically) • Manager encouragement to engage in JC as part of professional responsibility
Logistical administration of JC is established (time, venue, recurrent booking)	Enablement	• Allocate a set time, use of regular room and time to reduce clashes • Tie in meeting with another meeting,
Manager expectation holds staff to account to prioritise JC attendance	Coercion Persuasion	• Departmental leadership to advocate and value JC attendance and see as core business
Team culture values EBP	Modelling	• Ensure consistent message of value of EBP via members & managers, including new starter orientation
Increased participation by all JC members during group discussion	Modelling	• Facilitator to encourage participation from all group members during group appraisal, where possible have face to face rather than VC to facilitate interaction
Support from other clinicians in team	Enablement	• Regularly review topic choice to check relevance of topic to current practice
Capability		
Awareness of how to access Library & academics	Education	• Ensure Librarians available and engaged, to meet with JC and raise awareness of services available to JC members
Knowledge from academic needs to be pitched at right level	Enablement	• Academic must be skilled in ascertaining and monitoring learners in order to pitch information at a level appropriate to the skills and need of the JC members
JC members guided to choose topics relevant to all members	Education	• Provide support or education regarding how to prioritise topics and integrate into practice (i.e., relate to QI)
Skill development (e.g., in critical appraisal, EBP and stats training, statistics, identifying study design)	Training	• Access to regular training opportunities to ensure all staff have access to basic EBP training as they join a JC • Academic mentor attends initially to facilitate session then assists JC portfolio holders to facilitate using “cognitive apprentice model” (particularly upskill in facilitation of club) • Resources to support interpretation of study design and selection of CASP appraisal tool • Through building depth of clinician skill, wider numbers of JC members will have opportunity to build skills and confidence in application of skills
Access to academic or EBP-skilled clinician	Modelling Enablement	• Academic or skilled clinician available to support JC members in provision of relevant knowledge on-demand. • By providing direct guidance learner can be supported to move from peripheral to full participation. Guide must be able to ascertain readiness of the learner and be monitoring the learner’s development.

## Discussion

It is feasible for JCs to continue the general TREAT format within the allied health clinical setting; however, several contextual factors influenced sustainability. Interviews with participants provided insight into which evidence-based components of the TREAT format may be most easily sustained, as well as practical strategies which may promote longer term sustainability of the TREAT JC format within allied health.

JC participation and sustainability are not only impacted by individual factors such as clinician time and skill, as reported elsewhere [5], but other team, service and organisational factors including leadership culture and manager accountability, EBP experience within the team, and consistent staffing. The influence of leadership and manager accountability on JC participation has been reported in the medical literature [17] and elsewhere in regards to promoting EBP in health professionals [2, 18, 19]. The present study highlights the positive and potentially negative impact that leadership can have on accountability and valuing attendance at JCs as well as on a team’s EBP culture in general.

While staff changes are commonplace within the allied health workforce, their impact on JC participation or sustainability has not been reported. In the present study, staff changes may have resulted in clinicians being less motivated to continue the TREAT format, having not experienced the benefits of the format as reported by other clinicians. Indeed, while not a specific question in the interviews, in the present study clinicians frequently shared their positive perceptions of the TREAT format including its structure and ability to enhance knowledge and skills in comparison to previous formats. This is consistent with previous research which revealed clinicians were significantly more satisfied with the TREAT format compared to the standard format [10].

The use of behaviour change theory may assist in further understanding how barriers and enablers influence long term sustainability of a structured JC format and how to best implement the intervention in the future. As depicted in Fig. 1, key factors relating to opportunity, motivation and capability of Michie’s COM-B model [15] are needed to promote the long-term implementation of the JC format. While it has been suggested that increasing skills of JC members is important for sustainability in a previous study of a nursing JC in the ICU setting [20], our findings revealed that factors influencing motivation as well as opportunity are equally as important for sustainability. Indeed, a combination of factors influencing capability, opportunity and motivation are needed to promote sustainability. For example, the two JCs within our study which sustained the format both had a consistent group of staff attending the JC and a supportive EBP culture, thereby enabling more opportunities for greater ownership of the club which motivated them to attend, and share skills within their team to enhance capability. The factors outlined in Fig. 1 therefore may potentially be useful predictors in determining whether a JC is likely to be sustained or not, and it may be important to consider implementation strategies addressing these areas.

### Limitations

Due to staff movement, representation from one of the JCs in the focus group was from only one clinician. While we used a qualitative method to gather perceptions of sustainability and adaptations to the TREAT format, we acknowledge that additional objective measure related to treatment fidelity and adaptions may have also have been useful to support clinician reports. Participants were also from one health service and their experiences may not reflect experiences within other health service contexts. As all JCs recruited in our setting were predominately comprised of allied health professionals, it is unclear what impact participation of other professionals (i.e., medical and nursing) may have on influencing sustainability.

### Implications for research

To date, only one controlled trial has been undertaken evaluating JCs within allied health, further research into their contribution to EBP skills and clinical practice is warranted, with consideration of described implementation strategies in the present study. Also, further research to investigate the relative contributions of the different components of effective JCs would be helpful. Research within different geographical settings and contexts would also contribute to the understanding of factors which influence implementation and sustainability of JCs, as well as monitoring long term effects. Future studies of sustainability may also want to include more objective measures of observation or auditing to monitor treatment fidelity and adherence over the long term [13]. As all JCs recruited in our setting were predominately comprised of allied health professionals, it is unclear what impact participation of other professionals (i.e., medical and nursing) may have on influencing sustainability.

### Implications for practice

Teams planning to implement a JC should consider implementation strategies which are informed by behaviour change theory to enhance sustainability, as outlined in Table 6. These use a combination of both bottom up and top down strategies to enhance motivation, capability and opportunity. For example, to enhance clinician ownership and subsequent motivation as well as clinician capability rather than an academic mentor facilitating sessions and asking clinicians to continue the format independently, the use of a cognitive apprenticeship model which according to workplace learning literature describes the facilitation of learning through active participation in authentic learning experiences and uses legitimate peripheral participation may be useful for future implementation [21] could be adopted. Using this approach, academic mentors train other clinicians in how to facilitate the session, be accessible for support as required to maximise the capability of clinicians within the team, and encourage their ownership in the format. Further training in EBP should also be considered to supplement the JC, and may also include increasing awareness of library services. Implementation of the JC must also take into consideration service or team level factors, including considering training staff who are less likely to rotate (i.e., permanent senior staff) and engaging with management to foster a culture which values EBP and JC attendance. These strategies for sustainability should be considered early when implementing JCs to have maximum effect [22]. Criteria such as the APEASE which stands for assessing the Affordability, Practicability, Effectiveness, Acceptability, Side effects/safety, and Equity of suggested implementation strategies may be helpful when applying these to other contexts [23].

## Conclusion

JCs do not occur in controlled laboratory conditions but in complex healthcare environments with competing organisational and clinical demands. The present research suggests that the structured TREAT JC format is positively perceived by clinicians and has potential to be sustained within a busy clinical setting. The long-term sustainability of the format is heavily dependent on both individual and service level factors and a balance of implementation strategies that enhance opportunity, motivation and capability for sustainability. Planning how clinicians can be engaged to take ownership and build their own capability from the commencement of the JC is important, as well as consideration of how individual components can be implemented within local contexts and team cultures.

## Additional file

Additional file 1: Interview guide. (DOCX 14 kb)

## Abbreviations

EBP: Evidence based practice
GCH: Gold Coast Health
JC: Journal Club
TREAT: Tailoring Research Evidence and Theory
