# Supplementary material for: Factors that influence the sustainability of structured allied health journal clubs: a qualitative study
Source: BMC Med Educ. 2019 Jan 3;19:6. doi: 10.1186/s12909-018-1436-3 (PMC6318909; doi:10.1186/s12909-018-1436-3)
Supplement: Supplementary file 1 — Interview guide. (DOCX 14 kb) [file 12909_2018_1436_MOESM1_ESM.docx]

**Interview Guide**

1. **How would you describe your experience with participating in the TREAT journal club format over the last 6 months?**
2. **How is your experience of TREAT journal club been without the formal facilitation of the academic mentor?**
3. **Are there any adaptations you have made to the original TREAT journal club format?**
4. {Facilitator to show list of core components of TREAT club to members} **Which two or three of these components have been the easiest to sustain within your journal club?**

(for each of the listed components)

- 1. **What do you think has helped to sustain this component in your journal club?**

1. **Which two or three components have been the most difficult to sustain within your journal club and why?**

(for each of the listed components, further prompts)

- 1. *Do you think clinician’s knowledge or skills within your journal club may have impacted on implementing this component?*
  2. *Do you think anything related to your environment or access to resources may have impacted on this?*
  3. *Do you think journal club members were motivated to implement this component (why/why not)*

1. **Do you have any other suggestions things that may help to implement or embed the TREAT format?**

Or are there any other suggestions for improvement to the format? (i.e., that would make it easier to implement)

1. **What does your journal club plan to do in the next 6 months?**
2. **Is there any other feedback or comments you wanted to share?**

| **Components of TREAT format** |
| --- |
| - **Goal setting session at beginning of 6 month trial to prioritise topics** |
| - **Club at same time and place each month** |
| - **Articles circulated prior to session** |
| - **Access to librarian support** |
| - **Powerpoints and handouts given about topics related to journal club,** |
| - **Researcher/Academic available during session and between for support** |
| - **Used structured CASP tools for critical appraisal** |
| - **Group discussions encourage all members to actively appraise articles** |
| - **Opportunity to discuss how evidence can be applied in clinical setting** |
| - **Minutes being undertaken during session by a scribe and action items to be followed up** |
| - **Facilitator helping to guide discussion** |
| - **Food optional at journal clubs** |
